# The hCMEC/D3 cell line as a model of the human blood brain barrier

**DOI:** 10.1186/2045-8118-10-16

**Published:** 2013-03-26

**Authors:** Babette Weksler, Ignacio A Romero, Pierre-Olivier Couraud

**Affiliations:** 1 Weill Cornell Medical College, New York, NY, USA; 2 Department of Life, Health and Chemical Sciences, Open University, Milton Keynes, U.K; 3 Inserm, U1016, Institut Cochin, Paris, France; 4 CNRS, UMR8104, Paris, France; 5 Université Paris Descartes, Sorbonne Paris Cité, Paris, France

**Keywords:** Blood–brain barrier, Immortalized cell line, Brain endothelium, In vitro model

## Abstract

Since the first attempts in the 1970s to isolate cerebral microvessel endothelial cells (CECs) in order to model the blood–brain barrier (BBB) *in vitro*, the need for a human BBB model that closely mimics the *in vivo* phenotype and is reproducible and easy to grow, has been widely recognized by cerebrovascular researchers in both academia and industry. While primary human CECs would ideally be the model of choice, the paucity of available fresh human cerebral tissue makes wide-scale studies impractical. The brain microvascular endothelial cell line hCMEC/D3 represents one such model of the human BBB that can be easily grown and is amenable to cellular and molecular studies on pathological and drug transport mechanisms with relevance to the central nervous system (CNS). Indeed, since the development of this cell line in 2005 over 100 studies on different aspects of cerebral endothelial biology and pharmacology have been published. Here we review the suitability of this cell line as a human BBB model for pathogenic and drug transport studies and we critically consider its advantages and limitations.

## Review

### Derivation and selection of hCMEC/D3 cells

The hCMEC/D3 cell line was derived from human temporal lobe microvessels isolated from tissue excised during surgery for control of epilepsy. The primary isolate was enriched in CECs. In the first passage, cells were sequentially immortalized by lentiviral vector transduction with the catalytic subunit of human telomerase (hTERT) and SV40 large T antigen, following which CEC were selectively isolated by limited dilution cloning, and clones were extensively characterized for brain endothelial phenotype [1].

The hCMEC/D3 cells form a contact-inhibited monolayer of elongated cells on collagen type I or type IV. They do not show adhesion-independent growth in soft agar but form capillary structures in Matrigel, a characteristic property of cultured endothelium. They were reported to have an apparently normal diploid human karyotype [1], although a high-resolution multicolor fluorescence *in situ* hybridization (FISH) approach revealed a more complex karyotype at high passages than initially thought [2]. In addition, they stain positively for endothelial markers including CD34, CD31, CD40, CD105, CD144 (VE-cadherin) and von Willebrand factor, but not for CD36, which is absent from brain endothelium. They maintain stable growth and endothelial marker characteristics, at least until the 35^th^ passage.

## The ‘transport barrier’ in hCMEC/D3 cells

Optimal culture conditions are essential for a brain endothelial phenotype with mature adherens junction (AJ) and tight junction (TJ) protein expression and a strong permeability barrier function. Full differentiation associated with expression of CEC markers like TJ proteins requires cellular quiescence, achieved either by removal of key growth factors and/or by exposing cells to shear stress under flow (see below). Substrates for cell growth may also contribute to differentiation. For example, hCMEC/D3 monolayers display five-fold higher concentrations of TJ proteins on transwell filters than on plastic coverslips; in the same vein, we shall describe below the hCMEC/D3 response to activation of Wnt/β- catenin signaling, known to induce BBB formation during fetal development.

### Expression of junctional proteins

In the context of endothelial cell junctions, hCMEC/D3 cells are positive for junction-associated Ig-like proteins such as PECAM-1 and JAM-A, for AJ and TJ structural proteins such as VE-cadherin, claudin-3,-5 and occludin as well as for scaffolding proteins such as beta catenin and zonula occludens (ZO)-proteins-1 and 2 [1,3]. The small G-protein Gαi2, suggested as a TJ-associated protein, was indeed identified as a partner of claudin-5 and its presence was necessary for TJ formation in hCMEC/D3 cells [4]. Expression of claudins and occludin at intercellular junctions is best observed when the cells are confluent, treated with anti-inflammatory steroids such as hydrocortisone, anti-oxidant agents such as resveratrol, or the Wnt/β-catenin signaling activator, lithium chloride (LiCl). The Wnt/β-catenin pathway acts in hCMEC/D3 cells to induce/enhance the BBB phenotype by increasing expression of claudins as demonstrated in primary mouse CECs [5]. Similarly, all growth factors, particularly vascular endothelial growth factor (VEGF), should be removed from the culture medium with the exception of basic fibroblast growth factor (bFGF) to enhance expression of junctional proteins. The hCMEC/D3 cells express other newly identified junctional proteins such as annexins-1 and -2, which also appear to be important for maintenance of TJ integrity [6].

Comparison of transcriptional profiles of hCMEC/D3 cells and primary human CEC with freshly isolated mouse CEC confirmed the expression by hCMEC/D3 cells of a substantial number of genes expressed by brain endothelium, but showed lower expression of claudin-5, occludin, JAM-2, glut-1 and the insulin receptor [7]. The authors concluded that in order to attain a mature brain endothelial phenotype, other cell types present in the neurovascular unit (e.g. astrocytes, pericytes) regulate gene expression by CEC, suggesting that a more complex *in vitro* model might be required to fully mimic the BBB. Alternatively, in line with the aforementioned differentiating action of the Wnt/β-catenin pathway, complementing the hCMEC/D3 culture medium with astrocyte- and/or pericyte-derived soluble factors might be sufficient for further differentiation towards a BBB phenotype.

### Restricted permeability to paracellular tracers

Monolayers of hCMEC/D3 show restricted permeability to lucifer yellow (LY: a low molecular weight paracellular diffusion marker) and to many hydrophobic and hydrophilic low molecular weight drugs which correlate with *in vivo* permeability coefficients as demonstrated by Weksler *et al*[1] and confirmed by Poller *et al*[8]. They also show a restricted permeability to low and high molecular weight dextrans that is similar to primary CECs and lower than non-cerebral endothelium (e.g. human umbilical vein endothelial cells, HUVECs), particularly under conditions of flow [9]. Indeed, for compounds of MW>4000, the permeability profile is very similar to that of bovine and porcine CECs, hitherto the best characterized *in vitro* BBB models. As discussed above for TJ protein expression, the permeability barrier function is maximized in the presence of LiCl and corticosteroids (or resveratrol): in these conditions, the permeability coefficient (Pe) for LY, is: 1.55 +/- 0.16 10^-3^ cm/min. For comparison, Pe values for 4 kDa- and 70 kDa-dextrans are: 0.72 +/- 0.07 10^-3^ cm/min and 0.09 +/- 0.01 10^-3^ cm/min, respectively.

Conversely, stress conditions and extracellular stimuli have been shown to increase paracellular permeability of hCMEC/D3 cells via signaling pathways such as JNK, PKC or NFκB. These include mannitol treatment, oxygen and glucose deprivation (OGD) and pro-inflammatory cytokines such as TNFα and chemokines such as CCL2. Cowan *et al*[10] examined the effects of OGD under static conditions in hCMEC/D3 cells. They observed a reversible increase in monolayer permeability to dextran after 1 h of OGD without cytotoxicity, but permanent changes in monolayer permeability and marked cytotoxicity after 12-24 h. The acute permeability changes involved the generation of nitric oxide and could be prevented by blocking inducible nitric oxide synthase. Other studies have demonstrated that cytokines/chemokines increase the paracellular permeability of hCMEC/D3 cells to dextrans via different mechanisms [11]. With pro-inflammatory stimuli, ZO-1, occludin and claudin-5 expression levels are decreased [11,12], whereas JAM-A translocates away from tight junctions, without any expression changes [13]. The chemokine CCL2, which is elevated during CNS inflammation and is associated with endothelial dysfunction, transiently induces Src-dependent disruption of hCMEC/D3 AJs, translocation of β-catenin from the AJ to PECAM-1, and increases surface localization of PECAM-1 [14].

In brief, these studies illustrate the usefulness of the hCMEC/D3 model for unraveling the regulatory mechanisms of junctional integrity and BBB permeability in pathological conditions (for a review see [15]).

### Transendothelial electrical resistance (TEER)

Although the TEER of human cerebral microvessels has not been directly determined, it is widely accepted that mammalian systems such as the rat show high TEER values well above 1,000 Ω·cm^2^, a characteristic of the BBB *in vivo*[16]. However, TEER values above 1,000 Ω·cm^2^ are difficult to achieve in cultured CEC *in vitro* and this is particularly true for cell lines compared to primary cultures. Under static culture conditions, hCMEC/D3 monolayers develop only a low to medium-level TEER (around 30-50 Ω·cm^2^) in various reports. Interestingly, higher TEER values close to 300 Ω·cm^2^, were observed in the presence of hydrocortisone, probably due to the modulatory activity of corticosteroids on the expression of TJ proteins such as occludin and claudin-5 [12]. Another strategy targeted at increasing TEER values in hCMEC/D3 cells has involved co-culture with other cell types forming the neurovascular unit, as suggested above. In a recent paper, co-culture of hCMEC/D3 cells with astrocytes from different brain regions evoked a significant TEER increase from 30 to over 60 Ω·cm^2^[17]. In both monocultures and co-cultures of hCMEC/D3 with astrocytes, TEER values increased from baseline over an interval of 5 days, presumably due to TJ maturation with time. By far the most promising method to increase hCMEC/D3 cell TEER values has been exposure to flow-based shear stress. Indeed, in hCMEC/D3 monolayers subjected to pulsatile flow after seeding in a capillary cartridge system, the TEER was reported to rise to 1000-1200 Ω·cm^2^, then to rapidly drop following flow cessation [8]. Co-culture with astrocytes did not induce any further increases in TEER values in this flow-based model suggesting that, at least *in vitro*, shear stress may be a more critical factor in inducing a mature barrier phenotype than interactions with other cell types.

## The ‘transport’ barrier in hCMEC/D3 cells

Efflux and trans-cellular transport systems expressed by CECs are key factors for studying and predicting interactions of drugs at the BBB; an adequate pattern of transporter expression thus constitutes a prerequisite for suitable *in vitro* human BBB models.

### Expression, function and regulation of ABC transporters

hCMEC/D3 cells express functional efflux transporters (known as ABC transporters because they contain ATP-binding cassette(s) for active transport), typical for brain endothelium, as observed in freshly isolated human brain microvessels: these include P-glycoprotein (P-gp or MDR1 or ABCB1), breast cancer resistance protein (BCRP or ABCG2), and multidrug resistance-associated proteins (MRP) -4 and -5 (or ABCC4 and 5) [18]. In addition, hCMEC/D3 cells express MRP-1, as previously reported with primary human brain endothelial cells in culture, strongly suggesting that *in vitro* culturing may non-physiologically induce the expression of this gene [18]. Protein expression of P-gp/MDR1, MRP4, BCRP by hCMEC/D3 cells (grown on collagen-coated dishes) was further assessed by quantitative proteomic analysis [19], whereas no expression of P-gp/MDR1 was detected in HUVECs, used as reference non-brain EC. Interestingly, expression levels of P-gp, BCRP and MRP4 were similar in hCMEC/D3 cells and in isolated human brain microvessels [19]. Moreover, these key transporters are functional in hCMEC/D3 cells as efflux transporter inhibition studies invariably leads to elevated intracellular levels of their substrates [1,8,18]. In addition, P-gp expression is polarized to the apical membrane as previously demonstrated *in situ* in human brain microvessels and appears to be stable for at least 40 passages [20].

ABC transporter activity and/or expression levels may be modulated by extracellular stimuli. For example, Poller *et al*[21] noted that P-gp activity was not altered by TNF-α treatment, although P-gp expression levels increased after treatment. However, it is noteworthy that apparent increases in P-gp expression have been found in some cases to be due to selection of high Pgp-expressing hCMEC/D3 cells, for example, after exposure to potentially cytotoxic agents, and so may not reflect real increases in P-gp expression [22]. Substrates for P-gp can increase its level of expression and activity as demonstrated in hCMEC/D3 cells exposed to the HIV-1 protease inhibitors ritonavir and atazanavir, both substrates for P-gp. Inhibition of P-gp (but not of MRP-1) increased transport of these protease inhibitors. These drugs bind the xenobiotic receptor PXR which likely acts as a transcription factor for P-gp [23]. Concern about P-gp up-regulation during long-term administration of antiretroviral therapy, thus possibly blocking brain entry of these protease inhibitors (as well as of other therapeutic drugs) suggests that the hCMEC/D3 model may prove useful in designing newer antiretroviral therapies that use other means of crossing the BBB. Of interest, HIV-1 Tat can also lead to up-regulation of P-gp expression and hence contribute to decreased entry of antiretroviral therapy into the CNS [24].

In contrast to P-gp, BCRP expression and activity are decreased by inflammatory cytokines, in particular IL-1β and TNFα [21]. Conversely, agonists of the peroxisome proliferator-activated receptor alpha (PPARα) up-regulate BCRP in hCMEC/D3 cells, and can significantly decrease accumulation of drugs that are BCRP substrates (e.g. mitoxantrone). PPARα antagonists down-regulate BCRP in these CECs [25] suggesting new targeting strategies for either improving drug brain bioavailability or increasing neuroprotection. Along the same lines, BCRP was shown, using hCMEC/D3 cells, to mediate the transport of nifurtimox, an anti-trypanosomal drug [26]. These observations indicate that BCRP inhibitors potentially could improve the activity of anti-trypanosomal drugs and confirm that the hCMEC/D3 model is appropriate to test novel drugs.

### Influx transporters of the solute carrier family and receptor-mediated transcytosis

Brain endothelium is known to express a large number of membrane receptors and transporters that specifically control the blood-to-brain transport of nutrients, including insulin, transferrin and LDL receptors as well as glucose, amino-acids and organic ion transporters, all members of the solute carrier family (SLC) of transporters. Accordingly, hCMEC/D3 cells were tested for expression of these receptors and transporters by immunochemical analysis, RT-PCR and/or quantitative proteomic analysis. First, they were shown to express at a high level the glucose transporter Glut-1 and the transferrin receptor. Indeed, Glut-1 expression was found by quantitative proteomic analysis to be 15-fold higher in hCMEC/D3 cells than in HUVECs and similar to that of human brain microvessels [19]. Influx transporters such as the cation transporter OCT-1, and to a lesser extent OCT-2 and -3 are expressed and functional in hCMEC/D3 cells. OCT-1 is responsible for CEC uptake of the antiepileptic drug lamotrigine, a process blocked by the selective inhibitor prazosin [27]. Also, hCMEC/D3 cells express the neutral and cationic amino acid transporter (ATB^0^,^+^), which may be involved in the brain uptake of the anti-influenza compounds amantadine and rimantadine [28]. In addition, Carl *et al*[29] reported the expression by hCMEC/D3 cells of the monocarboxylate transporters SLC16A1 and SLC16A3 (MCT1 and MCT3), while little or no expression of SLC16A2 (MCT2) was noted. In agreement with these data, a high level of SLC16A1 expression at the protein level was detected by quantitative proteomic analysis of hCMEC/D3 cell extracts [19]. Regarding the proton-coupled oligopeptide transporter superfamily (POT, SLC15A) transporters, Carl *et al* also reported that hCMEC/D3 cells express both hPHT1 and hPHT2, while little to no expression of either hPepT1 or hPepT2 was observed, in line with previous data in the human BBB *in vivo*[29].

## The ‘metabolic’ barrier in hCMEC/D3 cells

The activity of drug-metabolizing enzymes, especially phase 1 cytochromes P450 (CYPs), might also indirectly control the cerebral uptake of compounds from the blood [30]. The aryl hydrocarbon nuclear receptor (AhR) was detected in hCMEC/D3 cells and dioxin (a ligand of AhR) treatment increased cytochromes P450 CYP1A1 and CYP1B1 over 20-fold [18]. Interestingly, CYP1B1 was previously identified as the major CYP in freshly isolated human brain microvessels [31], suggesting that the hCMEC/D3 model may be well adapted for further studies regarding the regulatory mechanisms of CYP1B1 expression by brain endothelium.

## Drug vectorization and trans-cellular transport

Numerous studies of liposomes and nanoparticles as vehicles for crossing the BBB while avoiding efflux transporters have utilized hCMEC/D3 cells. For example, Chattopadhyay *et al*[32] showed that solid lipid nanoparticles encapsulating atazanavir can circumvent P-gp efflux activity that usually limits uptake of the drug. Markoutsa *et al*[33] tested immunoliposomes bearing both a monoclonal antibody to the transferrin receptor (OX-28) and another isotype-matched monoclonal antibody linked to the lipid particles via a biotin-streptavidin technique, and showed that these structures were well taken up and transcytosed. These authors concluded that the hCMEC/D3 model was useful for particle transport studies. More recently, a combination of LDL receptor-targeted liposome-encapsulated doxorubicin and statins, known to increase LDL receptor expression, was shown to increase the drug delivery across hCMEC/D3 monolayers [34], suggesting a new concept of drug delivery to the brain. The toxicity of gold nanoparticles was evaluated in hCMEC/D3 compared to epithelial cells [35]. Sodium citrate on the particle surface but not particle size contributed to impaired viability and proliferation of endothelial cells, which internalized fewer nanoparticles than epithelial cells.

Single chain camelid antibody VHH fragments with anti-glial fibrillary protein (GFP) activity as well as fusion protein VHH- GFP were able to cross hCMEC/D3 monolayers as “fluobodies” [36]. Indeed, the same VHH crossed the BBB *in vivo* in mice and localized to astrocytes, showing for the first time that an antibody was efficiently able to penetrate the BBB and target resident cells in the brain.

## Interactions of immune cells with hCMEC/D3 cells

Although the CNS was originally considered an “immune-privileged site” because of the presence of the BBB and the apparent absence of lymphatic drainage, it is now well recognized that activated lymphocytes and monocytes do infiltrate the CNS by crossing the BBB and that neuroimmune diseases such as multiple sclerosis are characterized by massive perivascular infiltrates around brain microvessels. The hCMEC/D3 cell line provides a useful model for deciphering the modes of interactions between human brain endothelium and activated immune cells.

### Response of hCMEC/D3 cells to inflammatory mediators

The hCMEC/D3 cells respond to inflammatory stimuli by increasing paracellular permeability to tracers (see previous section) and are able to support adhesion and migration of leukocytes by increased expression of adhesion proteins like ICAM-1 and VCAM-1 [1]. They express functional cytokine and chemokine receptors such as TNFR1 and 2, IFNGR1 and CXCR1-5 and CCR3-6 [1,37]. Indeed, Fasler-Kan *et al*[38] demonstrated TNFα activation of NFκB signaling, whereas interferon gamma (IFNγ induced activation of JAK/STAT signaling pathways, and upregulated MHC Class I. In addition, secretion of chemokines by CECs may be an additional mechanism for modulating leukocyte extravasation. Furthermore, hCMEC/D3 cells secrete chemokines in a similar fashion to primary human brain endothelium both under basal conditions (CCL2 and CXCL8) or following stimulation by cytokines (CCL5, CXCL10, CX3CL1 or fractalkine) [39,40].

### Leukocyte adhesion to and transmigration across hCMEC/D3 cells

Monocytes adhere to activated hCMEC/D3 cells and migrate across the monolayer. The interaction between human monocytes and hCMEC/D3 cells involves the generation of reactive oxygen species (ROS), release of tissue-plasminogen activator (tPA) from the endothelial cells and a subsequent increase in permeability of the endothelial monolayer to large molecules (>150 kDa). Degradation of occludin appears to mediate the opening of endothelial-endothelial TJs [41]. Blocking the ERK1/2 pathway can partly reverse the monocyte-induced opening of monolayer TJs and impede occludin degradation. The same mechanism as demonstrated in the hCMEC/D3 model underlies brain changes in experimental autoimmune encephalomyelitis in the rat, a model of multiple sclerosis, as well as in rat monocytes and rat brain endothelial cells *in vitro*, suggesting that it is a generalized mechanism and may be pertinent in multiple sclerosis pathology. The same authors recently reported that a modulator of the sphingosine-1-phosphate (S1P) receptor, known to reduce inflammatory lesions in multiple sclerosis (FTY720P or Gilenya®), actually maintains hCMEC/D3 cells in a state of immune quiescence associated with decreased transmigration of monocytes [42]. This result further validates the hCMEC/D3 model for investigating the regulatory mechanisms of inflammation at the BBB.

Monocyte adhesion to hCMEC/D3 cells is enhanced by endothelial treatment with TNFα or IFNγ and can be inhibited by antibodies to the integrin VLA-4. A role for the junction-associated prion protein PrP^C^ in monocyte transmigration through brain endothelial cells was demonstrated with hCMEC/D3 cells, using either the U937 monocytic cell line or fresh primary blood monocytes: antibodies to the prion protein inhibited monocyte transmigration across the endothelial layer, whereas anti-PECAM 1 antibodies had no effect [43]. This inhibition was also observed with mouse primary brain EC and with a rat brain endothelial cell line, suggesting, as above, a mechanism common to brain endothelium from several species.

Bahbouhi *et al*[44] used hCMEC/D3 cells as a BBB model to compare adhesion and transmigration across CEC by peripheral blood mononuclear cells (PMBC) or purified T cells from multiple sclerosis patients versus PBMC or T cells from healthy individuals. They observed that PBMC migration is dependent on PSGL-1 and LFA-1 present on the PBMC. Both CD4+ and CD8+ T cells utilize these ligands to adhere to brain endothelium via P-selectin and VLA-4, respectively, and adherence can be blocked by anti-ligand antibodies. In multiple sclerosis, the frequency of CD4+ T cells that are PSGL-1+ is significantly greater than in healthy individuals; CD8+ cell populations were similar in both MS patients and controls. Transmigration of PBMC from multiple sclerosis individuals was enhanced across both resting and TNFα-activated hCMEC/D3 cells. The absolute transmigration was much greater across TNFα-activated hCMEC/D3 cells. Interestingly, PMBC from individuals treated with IFNβ a widely used first-line treatment of multiple sclerosis) had lower rates of transmigration and demonstrated lower LFA-1 levels.

Whether human neutrophils induce permeability changes in brain endothelium was studied by Joice *et al*[45] using hCMEC/D3 monolayers. This study was undertaken to understand whether neutrophil accumulation contributes to vasogenic edema in stroke. Untreated neutrophils applied to the hCMEC/D3 monolayers for 30 min actually decreased baseline permeability to low molecular weight (4 kDa) dextran by 53%, whereas neutrophils preactivated with TNFα, LTB4 or PMA (treatments that induced marked release of ROS) had no effect on baseline permeability. The authors then showed, in rats injected intracerebrally with human neutrophils, that very similar changes in brain vascular edema were seen. The authors concluded that the hCMEC/D3 model was useful in evaluating potential contributions to vasogenic edema.

## The hCMEC/D3 model for investigating Host-Pathogen Interactions

The hCMEC/D3 cell line has been widely used to model brain endothelium for investigating the molecular mechanisms of its interaction with and response to multiple human pathogens (viruses, fungi, bacteria and parasites) known to affect the CNS. Below are mentioned some of the most exciting results reported in this field.

### Retroviruses

Studies related to retroviral infection have concerned two pathogens, HTLV-1 and HIV-1. HTLV-1 infects hCMEC/D3 cells via their receptors for viral entry, Glut-1 and neuropilin-1, an observation that has been confirmed *in situ* in necropsy material from patients with TSP/HAM (tropical spastic paraparesis/human T-lymphotropic virus type-I-associated myelopathy) [3]. CEC infection leads to increases in paracellular permeability and TJ disorganization, probably via expression of the viral protein Tax. An additional mechanism leading to BBB disruption is via secretion of TNFα and IL1α by HTLV-1 infected T cells [46].

In the context of HIV-1, studies on hCMEC/D3 cells have focused on 1) mechanistic studies on HIV-1-induced BBB breakdown or 2) a model to investigate effects of anti-HIV therapeutics, particularly protease inhibitors, on BBB function (see previous section). For mechanistic studies, it has been demonstrated that HIV-1 and/or Tat protein induces disruption of claudin-5 and increases permeability of hCMEC/D3 cells in a similar fashion to effects on primary rodent BECs [47]. Tat-induced delocalization of ZO-1 from the membrane into the nucleus is mediated by Rho signaling and CREB [48]. In addition, Tat induces hCMEC/D3 cells into an activated inflammatory state by inducing increased expression of IL-1β, E-selectin, CCL-2, and IL-6 [49], an effect that is attenuated by PPARα and PPARγ agonists [50] via matrix metalloproteases [51]. As a result, HIV-1-infected monocytes, or Tat protein itself, have been shown to increase ICAM-1 expression and to favor transmigration of the infected monocytes across hCMEC/D3 cells by a mechanism that involved NFκB-induced release of MMP-9 [52].

HIV Tat also induces amyloid beta (Aβ) peptide accumulation in hCMEC/D3 cells which may contribute to its effect on BBB function [53]. Aβ accumulation and Tat-induced barrier dysfunction are lipid raft- and caveolae-dependent and involve caveolae-associated Ras signaling [54,55]. As mentioned above, Tat can also lead to up-regulation of P-gp expression and hence contribute to decreased entry of antiretroviral therapy into the brain [24].

### Cryptococcus

Adhesion to and penetration across a monolayer of hCMEC/D3 cells by the fungal pathogen *Cryptococcus neoformans* was demonstrated by Vu *et al*[56], who found that a large polysaccharide capsule on the fungus plus CD44, the hyaluronic acid receptor present on the hCMEC/D3 cells, were both important for the adherence of fungal particles to endothelial cells. Upon adherence of Cryptococci, the endothelial cells developed microvilli that attached to the fungi and appeared to aid in their transcytosis. Conversely, removal of hyaluronic acid or use of non-encapsulated organisms blocked adherence. The authors pointed out that although the TEER of the monolayers was low—about half that of primary brain endothelial cells—it was not further lowered by the adherence of Cryptoccocci and appeared to constitute a genuine barrier.

### Meningococcus

Although meninogocci (*Neisseria meningitidis*) are commonly carried in the nasal and oral mucosa of humans, direct meningococcal infection of the brain, a devastating illness, is fortunately rare. How meningococci enter the brain has long been poorly understood, but hCMEC/D3 cells used as a model of the BBB have importantly contributed to the elucidation of this mechanism. Adhesion of meningococci on hCMEC/D3 monolayers induces translocation of multiple endothelial membrane proteins, including ezrin, moesin, and actin to form honeycomb cortical plaques beneath the meningococcal colonies. Coureuil *et al*[57] observed that type IV pili present on pathogenic meninogocci recruited to the site of bacterial colonies the Par3/Par6/PKCζ endothelial polarity complex. This complex normally plays a pivotal role in the establishment of eukaryotic cell polarity and governs the formation of intercellular junctions; its translocation to these cortical plaques led to the formation of ectopic intercellular junctional domains at the sites of bacteria-endothelial cell interactions and depleted junctional proteins at endothelial cell-cell interfaces. This response of hCMEC/D3 cells resulted in the opening of intercellular junctions, thus permitting paracellular bacterial infiltration across the endothelial barrier. Coureuil *et al* further [58] explored the hCMEC/D3 model to ascertain the signaling pathway that recruits the cortical plaques to meningococcal colony sites. They elegantly demonstrated that meningococci “hijack” another endothelial physiological pathway through activation of β-adrenergic receptors by their Type IV pili, followed by activation of the scaffolding protein β-arrestin and the tyrosine kinase Src. Activation of this pathway favors endocytosis of phosphorylated VE-cadherin, a normal component of TJs, further opening up endothelial TJs. Of note, these authors recently reported that this pathway is also used by non-brain microvascular endothelial cells, but is clearly distinct from that used by epithelial cells [59].

### Plasmodium falciparum

Cerebral malaria, a common complication of *Plasmodium falciparum* infection particularly in children, is one of the most severe and often lethal manifestations of this common tropical disease. Induction of cerebral edema during cerebral malaria is among the most feared complications of this disease, yet the mechanisms are not well understood. The hCMEC/D3 cell line has provided an excellent *in vitro* model for studying the detailed interactions between *P*. *falciparum* parasites and brain endothelium. Jambou *et al*[60] evaluated the mechanism of *P*. *falciparum*-parasitized erythrocyte adhesion to hCMEC/D3 cells and showed for the first time that this process involved trogocytosis, the transfer of membrane material from one cell (malarial antigens on parasitized erythrocyte) to another cell (endothelial cell), followed by ingestion of the entire parasitized erythrocyte. These authors compared the hCMEC/D3 cell line with the HBEC-5i cell line and showed that the HBEC-5i line displayed a more activated phenotype when unstimulated, expressing much higher levels of ICAM-1, an important receptor in the interaction between parasitized erythrocytes and brain endothelial cells [60]. Blocking ICAM-1 or TNFα activation of endothelial cells prevented cytoadhesion of parasitized erythrocytes and their ingestion. More recently, hCMEC/D3 cells were used by Zougbede *et al*[61] to demonstrate that *P*. *falciparum*-parasitized red blood cells could alter BBB integrity also by a mechanism independent of cytoadhesion, namely, by induction of metabolic acidosis, which also resulted in opening TJs in the hCMEC/D3 monolayer, a process which also would favor development of cerebral edema.

## The hCMEC/D3 model for investigating neurodegenerative diseases

It is now well recognized that brain endothelium dysfunction likely contributes to the progression of several neurodegenerative diseases, initially considered as purely due to neuronal alterations, like Alzheimer’s or Parkinson’s diseases. The hCMEC/D3 model has been widely used to study the toxic effects of Aβ peptides on brain microvasculature in the context of Alzheimer’s disease. Aβ 1-40, the most abundant toxic Aβ peptide around blood vessels, was shown to increase hCMEC/D3 monolayer permeability, in the absence of cytotoxic effects, via the down-regulation of the TJ protein occludin, without changing levels of claudin-5 or ZO-1 [62]. The Aβ 1-40 effect on permeability could be prevented by inhibiting JNK or p38MAPK, suggesting that these signaling pathways represented a possible therapeutic target in the treatment of Alzheimer’s disease.

Aβ peptides have been shown to decrease the activity of efflux transporters in hCMEC/D3 cells [63]. Indeed, when hCMEC/D3 cells were exposed to Aβ peptides, P-gp mRNA and protein levels decreased through down-modulation of the Wnt/β-catenin signaling pathway (by decreasing β-catenin level and increasing DKK-1, an endogenous Wnt signaling inhibitor). These changes were reversed by administration of Wnt3a. The effect was specific for P-gp, as MDR4 and BRCP were not affected in these studies.

The hCMEC/D3 cell line was used to study cerebral amyloid angiopathy (CAA), an age-associated hemorrhagic condition commonly found in sporadic as well as some familial types of Alzheimer’s disease. Fossati *et al*[64] observed that Aβ peptides induce caspase-mediated mitochondrial dysfunction, then apoptosis in hCMEC/D3 cells; Aβ peptides bearing familial CAA mutations were more toxic to CEC than wild type Aβ peptides. Apoptosis of hCMEC/D3 cells was associated more with oligomeric peptide forms than with amyloid fibrils, a finding consistent with increasing evidence that oligomers of Aβ rather than the precipitating fibers are the most neurotoxic form. Similarly, hCMEC/D3 cells were used to evaluate the contribution of metalloproteases to the pathogenesis of CAA [65]. When hCMEC/D3 were exposed to Aβ peptides, the cells increased both production and enzymatic activity of MMP2 which in turn degraded Aβ peptides to Aβ 1-16 C-terminal fragments resulting in decreased CEC apoptosis. Conversely, silencing MMP-2 led to further Aβ 40/42-induced mitochondrial dysfunction and increased apoptosis of hCMEC/D3 cells. Thus, MMP2 may represent a potential vasoprotective and neuroprotective response of the brain vasculature.

Finally, the hCMEC/D3 cell line has also been used to investigate Aβ clearance mechanisms from the CNS to prevent both neurotoxic and vasculotoxic effects. Indeed, a first report on hCMEC/D3 cells showing that Aβ is selectively effluxed when present on the luminal, but not the abluminal side [66] has been also confirmed in primary bovine CEC models [67] suggesting that P-gp may act as a protective mechanism against plasma Aβ but not participate in the clearance of brain Aβ although its relevance *in vivo* remains to be determined.

## Advantages and limitations of hCMEC/D3 cells

In summary, results from various laboratories worldwide indicate that hCMEC/D3 cells retain the expression of most transporters and receptors expressed *in vivo* at the human BBB, including MDR1, BCRP, MRP4, transferrin receptor, insulin receptor, Glut-1; they also express metabolizing enzymes and TJ proteins, as expected.

Relatively few alternative models of the human BBB have been proposed, either as primary human CEC or cell lines. The following table (Table 1) summarizes other human CEC lines that have been used within the last decade. In contrast to the hCMEC/D3 cell line, most of them have been only minimally characterized. This strengthens the conclusion that the hCMEC/D3 cell line constitutes a unique model for investigating the biology of human brain endothelium.

**Table 1:** Published immortalized human brain EC lines

**Cell lines**	**Immortalization procedure**	**Phenotype/limitations**	**TEER (Ω.cm**^**2**^**)**	**LY/sucrose permeability coefficient (10**^**-3 **^**cm/min)**	**Year (first pub)**	**Ref.**
**BB19**	HPV16 E6E7	Constitutive expression of VCAM-1, CD36	nd	1.35	1996	[68,69]
**HCEC**	SV40T	No permeability characterization	40±8	nd	2000	[70]
**HBEC-5i**	SV40T	No expression of CD31	180±10	nd	2006	[71]
**NKIM-6**	HPV16 E6E7	No expression of claudin-5 or occludin	100	nd	2007	[72]
**HBMEC-3**	SV40T	No information available	245±8	nd	2009	[73]
**TY08**	Temperature-sensitive SV40T	Low level of functional P-gp	37±5	Pe(inulin)=1.23	2010	[74]
**HBMEC/ciβ**	Temperature-sensitive SV40T and hTERT	Promising preliminary characterization	nd	2.6±0.4	2012	[75]

However, a recent publication elegantly described the preparation of human BBB ECs from induced pluripotent stem (iPS) cells or embryonic stem (ES) cells [76]. Indeed, pure brain EC populations were isolated following serial incubation of human iPS or ES cells first with medium favoring neural differentiation and later with medium favoring endothelial differentiation. These stem cell-derived CECs grew as pure cultures, exhibited brain TJ molecules and transporters and developed a high TEER, significantly higher than hCMEC/D3 cells. Although the reproducibility of this sophisticated approach remains to be confirmed, these results demonstrate that understanding the molecular mechanisms of BBB development and regulation permits efficient modeling of the human BBB *in vitro*. This new model displays excellent barrier characteristics and may, in the future, constitute for the pharmaceutical industry a key tool for investigating BBB permeability to candidate drugs.

## Conclusion

To date, the main advantage of the hCMEC/D3 cell line is that it represents a stable, easily grown and transferable population of human microvascular CEC that stably maintains a normal BBB phenotype. As illustrated above, it appears particularly well adapted for drug uptake and active transport studies, as well as for understanding the brain endothelium response to various human pathogens and inflammatory stimuli. Optimizing the TJ tightness of hCMEC/D3 cell monolayers still remains a major challenge in order to provide an *in vitro* model that might recapitulate all the characteristics of human BBB, encompassing permeability restriction with appropriate molecular exclusion and functional efflux and influx transport systems. As suggested above, culture under flow together with treatment with recently identified BBB modulators may greatly help design strategies for hCMEC/D3 optimization. The large network of laboratories currently working with this model worldwide actually constitutes a major asset for achieving this objective.

## Abbreviations

ABC-transporters: ATP-binding cassette transporters; AJ: Adherens Junction; BBB: Blood–brain barrier; BCRP: Breast cancer resistance protein; CYP: Cytochrome P-450; CECs: Cerebral endothelial cells; CNS: Central nervous system; LiCl: Lithium chloride; LY: Lucifer yellow; MDR-1: Multidrug resistance protein-1; MRPs: Multidrug resistance-associated proteins; OGD: Oxygen and glucose deprivation; P-gp: P-glycoprotein; PPAR alpha: Peroxisome proliferator-activated receptor alpha; SLC-transporters: Solute carrier transporters; TEER: Transendothelial electrical resistance; hTERT: Catalytic subunit of human telomerase; TJ: Tight junction.

## Competing interests

The authors declare that they have no conflict of interest.

## Authors’ contributions

BW, IAR and POC jointly analysed literature and wrote the review. All authors have read and approved the final version of the manuscript.
